# Non-invasive imaging of carotid arterial restenosis using 3T cardiovascular magnetic resonance

**DOI:** 10.1186/1532-429X-16-5

**Published:** 2014-01-08

**Authors:** Alistair C Lindsay, Luca Biasiolli, Steven Knight, Colin Cunnington, Matthew D Robson, Stefan Neubauer, James Kennedy, Ashok Handa, Robin P Choudhury

**Affiliations:** 1 Oxford Centre for Clinical Magnetic Resonance Research (OCMR), Cardiovascular Medicine Division, Radcliffe Department of Medicine, University of Oxford, John Radcliffe Hospital, Oxford OX3 9DU, UK; 2 Oxford Acute Vascular Imaging Centre (AVIC), Radcliffe Department of Medicine, University of Oxford, John Radcliffe Hospital, Oxford OX3 9DU, UK; 3 Investigative Medicine Division, Radcliffe Department of Medicine, University of Oxford, John Radcliffe Hospital, Oxford OX3 9DU, UK; 4 Nuffield Department of Surgical Sciences, University of Oxford, John Radcliffe Hospital, Oxford OX3 9DU, UK

**Keywords:** Atherosclerosis, Cardiovascular magnetic resonance, Carotid plaque imaging, Carotid arteries, Restenosis

## Abstract

**Background:**

Restenosis of the carotid artery is common following carotid endarterectomy, but analysis of lesion composition has mostly been based on histological study of explanted restenotic lesions. This study investigated the ability of 3T cardiovascular magnetic resonance (CMR) to determine the components of recurrent carotid artery disease and examined whether these differed from primary atherosclerotic plaque.

**Methods:**

50 patients underwent 3T CMR of both carotid arteries using a standard multicontrast protocol: time-of-flight (TOF), T1-weighted (T1W), T2-weighted (T2W), and PD-weighted (PDW) Turbo-Spin-Echo (TSE) sequences. 25 patients had previously undergone carotid endarterectomy (mean time since surgery 1580 days, range 45–6560 days), and 25 with primary asymptomatic atherosclerotic plaques served as controls. Two experienced reviewers analysed the multicontrast CMR images according to the presence or absence of major plaque features and assigned an overall classification type.

**Results:**

In patients with recurrent carotid disease following endarterectomy, the mean degree of restenosis was 51% (range 30–90%). Three distinct types of restenosis were identified: 5 patients (20%) showed CMR characteristics of fibro-atheromatous tissue, 11 patients (44%) had plaque features consistent with possible myointimal (fibromuscular) hyperplasia, and 6 patients (24%) had recurrent plaque suggestive of further lipid accumulation. Three patients (12%) showed evidence of post-surgical dissection of the carotid intima. Compared to primary atherosclerotic plaques, restenotic plaques were more likely to contain fibro-atheromatous tissue (p = 0.05) and smooth muscle (p < 0.01), and less likely to contain lipid (p < 0.01). Composition did not differ significantly between patients with early and late restenosis.

**Conclusions:**

As defined by CMR, restenotic lesions of the carotid artery fall into three distinct types and differ in composition from primary atherosclerotic plaques. If validated by subsequent histological studies, these findings could suggest a role for CMR in detecting high-risk (i.e. lipid-rich) restenotic lesions.

## Background

Restenosis of the carotid artery is common after carotid endarterectomy (CEA). Previous ultrasound studies have reported that restenosis can occur in approximately 10% of patients within the first year following CEA, with up to 37% of patients developing restenosis in the longer term [1], however more recent estimates have been lower [2]. Despite this, there is currently no consensus on the treatment of carotid restenosis. Apparently low rates of recurrent cerebrovascular events have led some centres away from repeat intervention [3]. Conversely, other authors have advocated that restenotic plaques should be treated by repeat carotid endarterectomy [4-6], or by carotid stenting [7-10].

Contributing to this management dilemma, histological studies performed to date have revealed that restenotic lesions of the carotid artery can show markedly different compositional features [11-15]. Cossman et al. identified that restenosis early after endarterectomy can largely be attributed to myointimal hyperplasia [16]. Importantly, these authors also highlighted that repeat surgery for this type of recurrent disease is technically difficult and unlikely to lead to a reduction in symptoms. In contrast, late recurrent disease of the carotid artery has been shown to be histologically more similar to primary atherosclerotic plaque; in particular, a large lipid core is more commonly seen in patients with symptomatic recurrent disease several years after endarterectomy [14]. These plaques may therefore present a greater clinical risk and may necessitate further carotid surgery, despite the increased risks and technical challenges associated with a repeat procedure.

However, tissue studies can give no information on restenotic plaques that are not deemed to need surgery. Multicontrast cardiovascular magnetic resonance (CMR) of the carotid artery is a well-established method of characterising atherosclerotic plaque morphology and composition [17], fibrous cap rupture [18], and lipid-rich core in native disease [19,20]. However, other than a recent paper describing three case reports [21], CMR has not previously been used to characterise restenosis of the carotid artery. Therefore the aims of this study were to apply CMR to characterise recurrent carotid plaque disease (restenosis), both early and late after endarterectomy, and to compare the features of recurrent carotid disease with primary atherosclerotic plaque in a control cohort.

## Methods

### Study population

50 patients were recruited from the database of the vascular ultrasound scanning laboratory at the John Radcliffe Hospital, Oxford. 25 patients had previously undergone carotid endarterectomy (mean time since surgery 1580 days, range 45–6560 days) and had subsequently been found to have restenosis (range 10–90%) at follow-up duplex ultrasonography. A further 25 patients with asymptomatic primary atherosclerotic plaque, matched for the degree of stenosis seen on ultrasound, were recruited as controls. Following an initial approach by letter, all patients gave written consent to participate in the study, which was approved by the regional ethics committee (Oxfordshire committee). All duplex ultrasound scanning was performed by a registered vascular scientist using a standard protocol to assess the common, internal, and external carotid arteries bilaterally. In addition to visual estimates of stenosis, Doppler flow velocities were used to estimate stenoses as follows: ≥130 cm/s, >50%; >180 cm/s, >65%; >230 cm/s, >70%; >300 cm/s, >80% [22].

### Scan protocol

All patients underwent 3T CMR using a standard multicontrast protocol, composed of time-of-flight (TOF), T1-weighted (T1W), T2-weighted (T2W), and PD-weighted (PDW) Turbo-Spin-Echo (TSE) sequences, to image 10 mm either side of the carotid bifurcation of the index artery. Black-blood cross-sectional images of carotid arteries were acquired with 4-channel phased-array carotid coils (Machnet, Netherlands) using Double-Inversion-Recovery (DIR) preparation and cardiac gating. Chemical shift selective fat saturation was used to suppress the signal from subcutaneous and perivascular fat. If necessary, a saturation band was positioned on the anterior region of the neck to reduce ghosting artefacts from breathing and swallowing. Gadolinium-based contrast (0.1 mmol/kg) was only used for restenosis patients who were able to tolerate the full scan protocol (TOF, T1, T2, and PD-weighted images) with ease and where the estimated Glomerular Filtration Rate (eGFR) was >60 ml/minute. Scan protocols were as follows: all T1W (TE = 14 ms, TR = 1 R-R interval and echo train length ETL = 9), T2W (TE = 89 ms, TR = 2 R-R and ETL = 15) and PDW (TE = 14 ms, TR = 2 R-R and ETL = 9) TSE images had slice thickness = 2 mm, FOV = 150 × 150 mm^2^ and matrix size = 320 × 320 (after zero-padding was 640 × 640 with pixel size = 0.234 mm); TOF angiography was acquired using 3D Fast Low Angle Shot (FLASH) with flip angle = 18°, TR = 72 ms, TE = 4.1 ms, FOV = 200 × 150 mm^2^, matrix size = 256 × 192 and slice thickness = 1 mm.

### Plaque analysis

Two experienced reviewers (A.L. and L.B.) - both of whom were blinded to the patients’ surgical status - analysed the multicontrast CMR images (excluding any post-gadolinium images) and recorded the presence or absence of the following plaque features: intra-plaque haemorrhage, calcium, lipid, and fibrous tissue as described in previous studies [19]. Calcification is characterized by very low proton density which produces hypointense signal compared to the adjacent sternocleidomastoid muscle on all weightings. Intra-plaque haemorrhage is identified by hyperintensity on T1W and TOF images and iso- to hyperintensity on T2W and PDW images (depending on the age of the haemorrhage). Fibrous tissue is hypointense on TOF and isointense on the other weightings. Lipid-rich necrotic core has a shorter T2 relaxation time than fibrous tissue and the sternocleidomastoid muscle [23] and so is recognisable by hypointense signal on T2W, whereas it appears isointense on all other weightings. The possible presence of myointimal hyperplasia was indicated by isointense signal relative to the surrounding tissues on all weightings [24]. Fibrous cap was assessed as defined by Hatsukami et al. [18]. An overall (predominant) phenotype (lipid-rich, fibro-atheromatous, hyperplasia) was assigned to each artery according to which plaque feature occupied the most slices. Plaques were then separated into two main groups depending on the time since carotid endarterectomy; ‘early’ restenosis related to those patients who had undergone surgery in the previous 4 years, while ‘late’ restenosis referred to patients who had surgery > 4 years previously.

### Statistical analysis

Continuous variables were compared using the unpaired Student *t* test where appropriate. All continuous variables were tested for normal distribution using the Shapiro-Wilk test, and if necessary analysis was performed using nonparametric tests (Mann–Whitney rank sum test or the Kruskal-Wallis tests, as appropriate). Differences in characteristics between groups were compared using chi-square analysis and Fisher’s exact test. A p-value < 0.05 was considered to be significant.

## Results

### Clinical data

The mean age of the group with recurrent carotid disease was 71 years and 4 of the 25 patients scanned (16%) were female. In the control group the mean age was higher (78 years, p = 0.01) and 8 patients were female (32%, p = 0.32) (Table 1). All patients had risk factors associated with atherosclerotic disease, and the degree of carotid stenosis as measured by ultrasound was similar in both patient groups (51% vs. 49%; p = 0.93). More patients in the recurrent carotid disease group were smokers (p = 0.03), however no other significant differences in the risk profiles between the two groups was noted.

**Table 1:** Comparison of clinical characteristics between the restenotic and primary atherosclerotic plaque (control) groups

	**Restenosis**	**Controls**	**p-value**
**Mean age (years)**	71	78	*0.01*
**Mean percentage stenosis**	51%	49%	*0.93*
**Female gender**	16%	32%	*0.32*
**Hypertension**	80%	72%	*0.74*
**Diabetes**	12%	28%	*0.29*
**Smoker**	84%	52%	*0.03*
**Previous myocardial infarction**	28%	16%	*0.50*
**Previous CABG**	16%	8%	*0.67*
**Treated lipids**	76%	68%	*0.75*
**Previous atrial fibrillation**	4%	8%	*1.00*

### Imaging of the carotid artery post-endarterectomy

**Figure 1:**
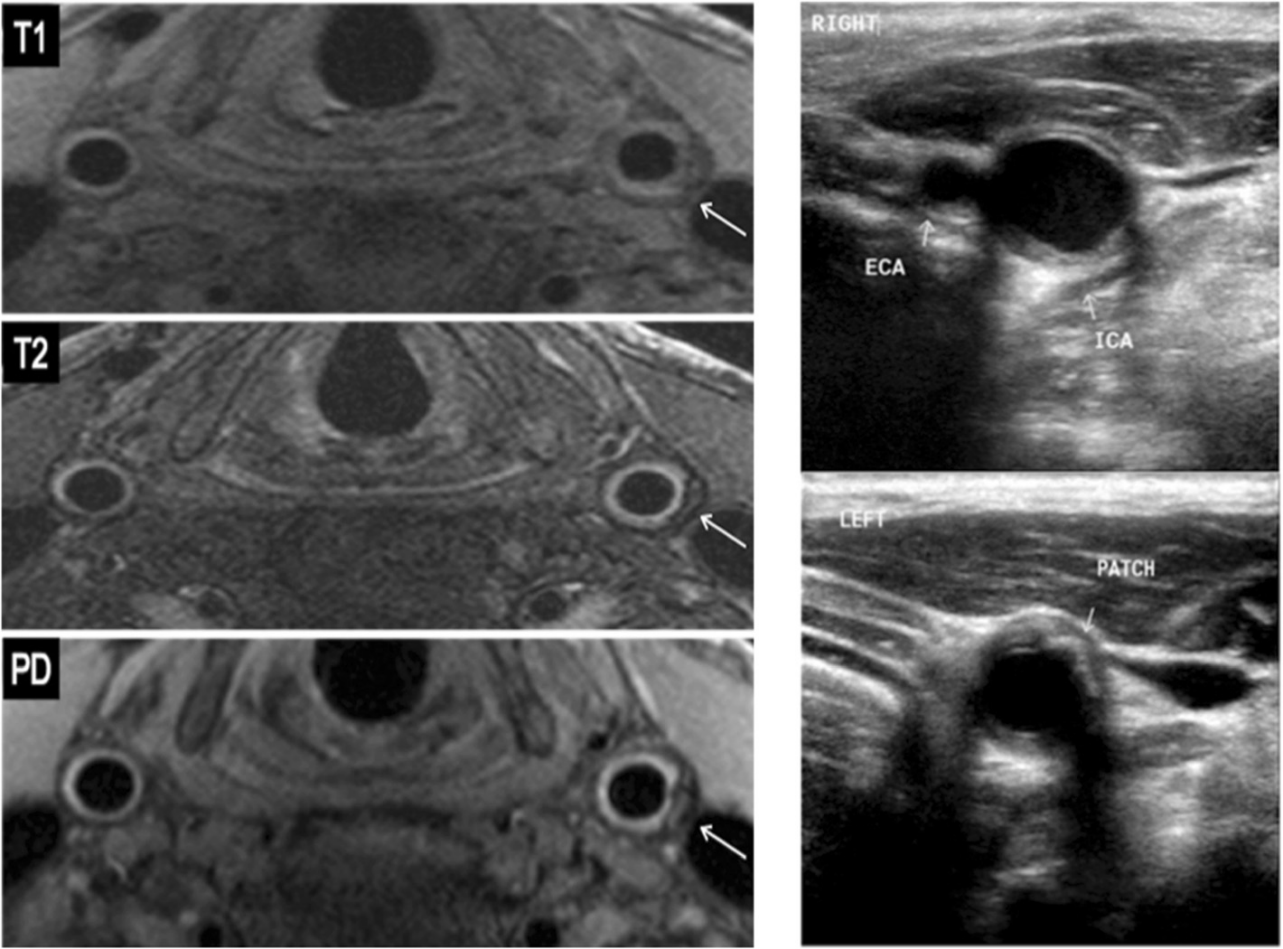
**Patient with bilateral carotid endarterectomy five and a half years previously (2038 days); T1, T2 and PD weighting of both common carotid arteries shown (left).** The right common carotid artery demonstrates a normal post-operative appearance, both on CMR and ultrasound (top right). However, a 30% restenosis of the left common carotid was noted on ultrasound (bottom right), which was seen as a crescent of predominantly low signal surrounding the artery on CMR (white arrows).

**Figure 2:**
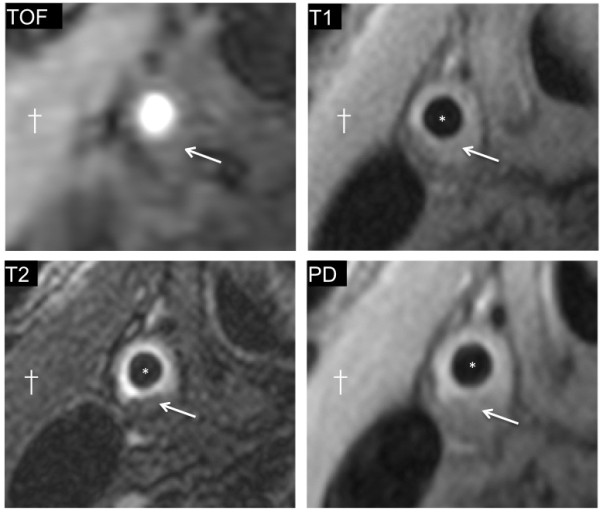
**Example of restenosis with the appearances of lipid reaccumulation.** 50% restenosis of the right common carotid artery is shown (*marks lumen). A crescent of plaque is seen that is isointense to the adjacent sternocleidomastoid muscle (†) on the T1W image (arrow). The same area shows low intensity signal on the T2W image (arrow), suggestive of lipid-rich plaque.

**Figure 3:**
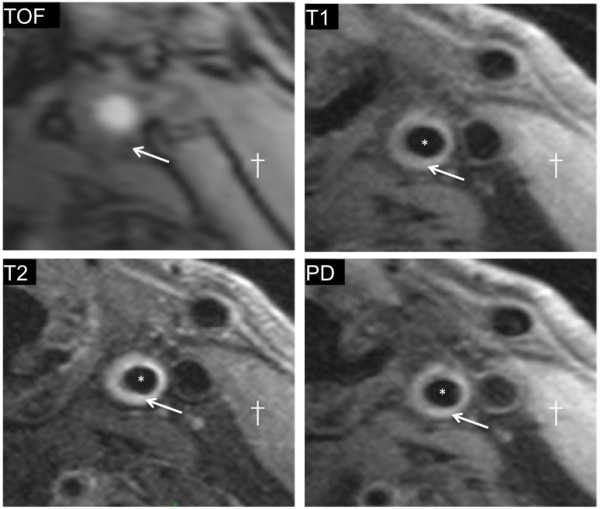
**Example of restenosis due to possible fibro-atheromatous narrowing.** 50% restenosis of the left internal carotid artery imaged 4 years after surgery is shown. The signal appears hypointense on TOF images (arrow), but isointense signal is seen from the tissue surrounding the lumen (*) on the T1W image (arrow); the signal is similar in intensity to that from the adjacent sternocleidomastoid muscle. Similarly, on PDW images (arrow) the signal appears to be isointense compared to sternocleidomastoid (†), suggestive of fibrous tissue deposition.

**Figure 4:**
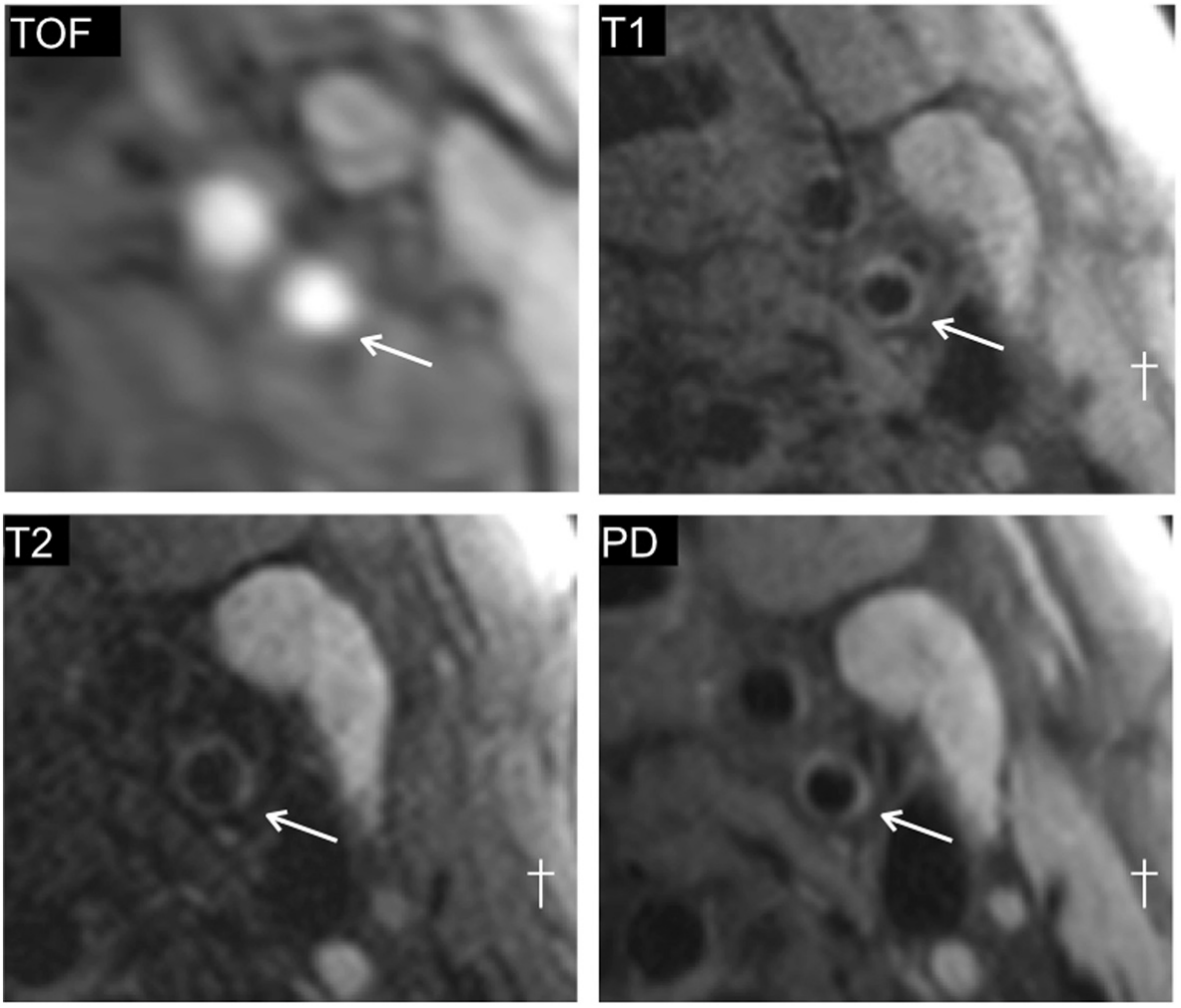
**Example of restenosis due to possible myointimal hyperplasia.** 65% restenosis of the left internal carotid artery 4 months following surgery is shown (*marks lumen, † marks sternocleidomastoid). The signal intensity seen from the arterial wall is comparable to that of the surrounding tissues on all four weightings.

**Figure 5:**
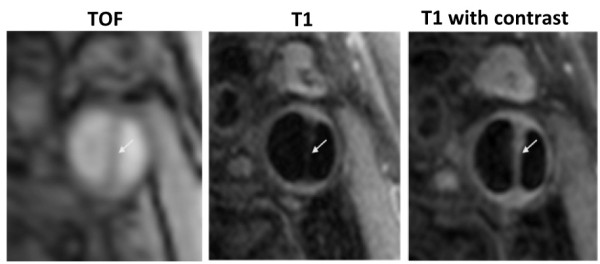
**Post-operative dissection of the carotid artery.** The patient had undergone endarterectomy several years before but remained symptom free, however a clear intimal flap is seen in all three images (arrow). The time-of-flight image on the left confirms the presence of a false lumen with blood flow. The T1W image (centre) shows the presence of an intimal flap, which shows an increase in signal intensity after the administration of gadolinium contrast (right), suggestive of a high degree of vascularity.

The mean degree of restenosis as measured by ultrasound was 51% (range 10-90%). Three patients with restenosis < 30% showed essentially normal arterial wall features on CMR (Figure 1). In the remaining patients, all with recurrent disease of the carotid artery causing 30% stenosis or greater, three distinct imaging patterns were noted. Six patients (24%) had recurrent plaque that was predominantly isointense to sternocleidomastoid on T1W and relatively hypointense on T2W, suggesting lipid accumulation (Figure 2). Five (20%) patients had recurrent carotid disease that was isointense with the sternocleidomastoid muscle on T1W, darker on TOF imaging, and isointense on T2W and PDW, suggestive of a predominance of fibro-atheromatous tissue in the arterial wall (Figure 3). Eleven (44%) patients showed evidence of arterial narrowing characterised by tissue that was largely isointense with the surrounding soft-tissues on all weightings, suggestive of possible myointimal hyperplasia (Figure 4). In addition, three patients (12%) showed evidence of post-surgical dissection of the carotid intima, as suggested by a line of tissue crossing the arterial lumen that showed increased signal intensity in response to gadolinium contrast (Figure 5).

### Comparison of recurrent carotid disease with primary atherosclerotic plaque

A comparison of the CMR features of recurrent carotid plaque compared to primary atherosclerotic plaque is shown in Table 2. While the presence of a thin fibrous cap, intra-plaque haemorrhage, and calcification were numerically more common in the primary atherosclerotic plaque group, these changes did not reach statistical significance overall. In contrast, fibrous tissue was more prevalent in the restenosis group (20% vs. 0%; p = 0.05). Furthermore, when compared to primary plaques, restenotic lesions were less likely to contain lipid (24% vs. 80%; p < 0.01), and more likely to show signs of myointimal hyperplasia (44% vs. 0%; p < 0.01). Comparison between plaques imaged early and late after endarterectomy revealed no statistically significant differences in plaque composition according to time since surgery. In particular, both myointimal hyperplasia (42% vs. 46%) and lipid (17% vs. 31%) were noted in restenotic lesions imaged both early and late after surgery.

**Table 2:** Comparison of plaque components between primary and restenotic plaques

	**Restenotic plaque**			**Primary plaque**	**P value (Primary vs. Restenotic plaque)**
	***All***	***Early***	***Late***		
		***(<4 years)***	***(>4 years)***		
Fibrous cap^*^	8%	8%	8%	24%	*0.25*
**Haemorrhage**	8%	0%	15%	24%	*0.25*
**Calcium**	0%	0%	0%	8%	*0.49*
**Lipid**	24%	17%	31%	80%	*< 0.01*
**Fibrous tissue**	20%	25%	15%	0%	*0.05*
**Myointimal hyperplasia**	44%	42%	46%	0%	*< 0.01*

* = No cases of fibrous cap rupture were seen.

## Discussion

This manuscript describes the use of 3T MRI to characterise carotid artery restenosis. The results identify three main types of recurrent carotid arterial disease following endarterectomy: lipid-rich, fibro-atheromatous, and a third subgroup with possible myointimal hyperplasia. Furthermore, this *in vivo* study confirms that the morphological features of recurrent carotid disease differ from those of primary atherosclerotic plaque. However, no significant difference was noted between the composition of restenotic plaque in those patients imaged early and late after surgery. The results suggest that characterisation of restenotic plaques by CMR may be of future clinical use.

Currently, no consensus exists for the management of restenosis of the carotid artery following carotid endarterectomy [25], and neither clinical follow-up nor surveillance imaging with ultrasound have been shown to improve clinical outcomes postoperatively [26]. Indeed, at present even the optimal timing of imaging of the carotid artery following endarterectomy is not agreed [27-29] although ultrasound follow-up is the most commonly used. However, although histological studies have shown the variable composition of restenotic lesions, ultrasound is often unable to provide detailed information on plaque composition; for example, in the early stages following surgery, recurrent plaque appears universally isoechoic [30]. A previous study of four patients undergoing popliteal artery angioplasty demonstrated the ability of CMR to show arterial remodelling, but did not give any information on restenotic plaque [31]. The results described here show that, using standard sequences, high-resolution 3T CMR can potentially give additional information on the composition of recurrent carotid plaque, which could allow for differentiation of relatively benign types of restenotic plaque (e.g. myointimal hyperplasia) from potentially more dangerous (e.g. lipid laden) plaque.

The relationship between traditional atherosclerosis risk factors and arterial restenosis remains a subject of debate, however smoking is considered to be the factor most predictive of restenosis [1]. This is in keeping with the findings of this analysis, which found that 84% of patients with restenosis had a history of smoking (Table 1). Age and sex have also been suggested as potential predictors of restenosis, and in this study patients with restenosis were found to be younger and more likely to be male (Table 1).

Previous histological studies examining the nature of carotid restenosis following repeat surgery have tended to be small. However, several such studies have suggested that the composition of recurrent carotid atherosclerosis varies according to the time that it develops. Whereas most cases of early restenosis (generally defined as restenosis within four years of surgery) show marked accumulation of smooth muscle cells and fibro-atheromatous tissue, restenosis that occurs after four years more closely resembles primary atherosclerotic plaque in that it generally contains far more lipid [14]. Although our study also noted that fibro-atheromatous tissue was more common in early restenosis, and lipid-rich plaque was more common in late stenosis, overall no statistically significant difference in plaque composition was noted between early and late lesions. There are several possible explanations for this observation. Firstly, histological studies are inherently biased towards patients in whom surgery was undertaken and excludes patients managed conservatively. This might be expected to lead to an over-representation of severe or symptomatic restenosis lesions, which are commonly lipid-rich. Secondly, histological studies have taken the time from operation to recurrence to make the distinction between ‘early’ and ‘late’ restenosis. In our cross-sectional study patients with apparently ‘late’ disease may have developed a recurrent lesion at an earlier stage, but with later recruitment. Lastly, while this study was able to detect differences in composition between native and restenotic lesions (below), a larger sample size may be required to detect more subtle within-group differences.

This *in vivo* study identifies important differences in the nature of restenotic - as opposed to primary – carotid atherosclerotic plaques that have only previously been described in histological examination of explanted tissue. Although many plaque features are common to both primary and recurrent disease, we found a higher prevalence of lipid-rich lesions in patients with primary atherosclerosis (80% vs. 24%, p < 0.01) compared to restenotic lesions (Figure 2). A separate type of restenosis showed appearances consistent with fibro-atheromatous disease (Figure 3). A third group of patients had significant restenosis characterised by a signal intensity that was similar to that of the surrounding tissues on T1, T2, and PD weightings (Figure 4), a finding which was not seen in the primary atherosclerotic plaque group (0% vs. 44%, p < 0.01). These signal characteristics were the most common finding in the early restenosis patients, a group in which myointimal hyperplasia caused by smooth muscle hypertrophy is known to be the most frequent cause of restenosis and calcification is rarely seen. Indeed, a histological analysis by Hellings et al. found that 100% of restenotic plaques occurring less than two years after endarterectomy contained smooth muscle cells and collagen; none contained calcium [14].

Lastly, three patients were noted to have developed post-endarterectomy dissection of the internal carotid artery. This complication of carotid artery surgery has previously been thought to be relatively rare [32], however it may be that current screening methods are poor at detecting its presence. It is important to be aware of this complication, as patients with asymptomatic internal carotid artery dissections may present with clinical symptoms months, or even years, later [33]. As such, the detection of this complication represents another potential benefit of using CMR post-endarterectomy.

## Conclusions

We have described the use of CMR to differentiate different types of carotid arterial restenosis *in vivo*. CMR of carotid restenosis is capable of giving detailed information on the composition of restenotic plaque and in addition may detect post-operative complications, such as internal carotid artery dissection. Further longitudinal studies with histological verification will be needed to confirm these findings and investigate the relationship between restenotic plaque appearances and the development of cerebrovascular events.

## Competing interests

There are no competing interests.

## Authors’ contributions

ACL recruited and imaged the study participants, analysed the data, and wrote the manuscript. LB assisted with imaging study participants, analysed the data, and revised the manuscript. SK assisted with imaging the study participants and revised the manuscript. CC assisted with imaging the study participants and revised the manuscript. MDR assisted with imaging and revised the manuscript. SN revised the manuscript. JK revised the manuscript. AH assisted with recruitment and revised the manuscript. RPC revised the manuscript. All authors read and approved the final manuscript.

## Authors’ information

Joint senior author: Ashok Handa and Robin P Choudhury.
